# A novel model of a biomechanically induced osteoarthritis‐like cartilage for pharmacological in vitro studies

**DOI:** 10.1111/jcmm.17044

**Published:** 2021-11-11

**Authors:** Katrin Sauerland, Amela Wolf, Manfred Schudok, Juergen Steinmeyer

**Affiliations:** ^1^ Institute for Pharmacology and Toxicology University of Bonn Bonn Germany; ^2^ R&D, Drug Metabolism & Pharmacokinetics Sanofi‐Aventis Deutschand GmbH Frankfurt Germany; ^3^ Laboratory for Experimental Orthopaedics Department of Orthopaedics University of Giessen Giessen Germany

**Keywords:** disease model, drug, matrix metalloproteinase inhibitor, metabolism, OA, Osteoarthritis, pharmacology

## Abstract

Excessive pressure or overload induces and aggravates osteoarthritic changes in articular cartilage, but the underlying biomechanical forces are largely ignored in existing pharmacological in vitro models that are used to investigate drugs against osteoarthritis (OA). Here, we introduce a novel in vitro model to perform pathophysiological and pharmacological investigations, in which cartilage explants are subjected to intermittent cyclic pressure, and characterize its ability to mimic OA‐like tissue reactivity. Mechanical loading time‐dependently increased the biosynthesis, content and retention of fibronectin (Fn), whereas collagen metabolism remained unchanged. This protocol upregulated the production and release of proteoglycans (PGs). The release of PGs from explants was significantly inhibited by a matrix metalloproteinase (MMP) inhibitor, suggesting the involvement of such proteinases in the destruction of the model tissue, similar to what is observed in human OA cartilage. In conclusion, the metabolic alterations in our new biomechanical in vitro model are similar to those of early human OA cartilage, and our pharmacological prevalidation with an MMP‐inhibitor supports its value for further in vitro drug studies.

## 1 INTRODUCTION

The common disease osteoarthritis (OA) ultimately results in a disintegration of the extracellular matrix of articular cartilage.^1^, ^2^ There are currently no drugs commercially available that have been clinically proven beyond doubt to prevent, slow down or even stop the progression of OA.^3^, ^4^ The discovery of new therapeutic targets and the development of new anti‐OA drugs is a lengthy and challenging activity for pharmaceutical companies and academic institutions.^4^, ^5^

Motion and mechanical stimulation are critical factors in the development and preservation of a healthy skeleton and normal joints.^6^, ^7^ In addition, the amount and types of certain mechanical parameters are critical in controlling the induction and progression of OA.^8^, ^9^, ^10^, ^11^, ^12^, ^13^ Joint failure can result from an asymmetry between both the mechanical impact and the catabolic activities that act on joints and the ability of the tissue to cope with such strains and repair the damage.^8^, ^13^, ^14^ Post‐traumatic OA, meniscectomy, hip dysplasia, joint malalignment and slipped capital epiphyses are examples of secondary OA, which may originate from atypical mechanical stresses that cause normal articular cartilage to degenerate.^15^, ^16^, ^17^, ^18^, ^19^, ^20^

Articular cartilage has distinctive features leading to exceptionally low friction and wear, and is the load‐bearing and viscoelastic tissue of joints.^21^ Chondrocytes sense their mechanical surroundings and respond accordingly by producing an extracellular matrix that is well able to dissipate mechanical loads, thereby protecting cartilage against injury.^9^, ^22^, ^23^, ^24^ Mechanosensitive ion channels, primary cilia and integrin receptors in the cell membrane sense changes in the streaming potentials, interstitial fluid, and hydrostatic and osmotic pressure that are induced by dynamic and static mechanical forces. These physical stimuli activate mechanotransduction pathways, which ultimately regulate transcription in chondrocytes.^9^, ^22^, ^23^

Using animal models is one way to practically examine the mechanisms involved in the initiation and progression of OA. With in vivo models, morphological changes of cartilage and joints can be reliably evaluated, but the cellular mechanisms that control these alterations are difficult to study and remain mostly unknown.^16^, ^25^, ^26^ In vitro cultures constitute a more direct approach to studying chondrocyte behaviour under controlled conditions. Various culture models have been developed, such as monolayer cultures, suspension cultures, chondron cultures, immortalized cell cultures, explant cultures and co‐culture systems.^25^, ^27^ Significant advantages of in vitro studies on metabolism are that the experiments can be controlled in a more precise manner and the experimenter can reduce the interindividual variability.

The phenotype of chondrocytes and the extracellular matrix is maintained in cartilage explants. In addition, the structural organization and interaction of the extracellular matrix with the chondrocytes are preserved in cartilage explant cultures. Furthermore, chondrocytes do not experience any enzymatic or mechanical stress being imposed on during cell isolation. Thus, an intact cell membrane with its receptors and other membrane glycoproteins is conserved. As such, cartilage explant cultures offer a more realistic method of investigating mechanical signals being transmitted through the sophisticated architecture of the tissue and the responses and functions of cells in their natural milieu.^16^, ^25^, ^28^

However, most organotypic cartilage explants are cultured at normal atmospheric pressure, whereby the chondrocytes are not exposed to compressive pressure or tensile strains. Since hyaline articular cartilage is exposed to compressive stimuli, especially in joints, some in vitro studies have already been carried out.^13^, ^14^ The effects of mechanical compression on cell proliferation, hypertrophy and apoptosis, as well as the biosynthetic response of the chondrocytes with regard to the extracellular matrix and catabolic enzymes were investigated.^13^, ^29^, ^30^ The mechanical devices used differ depending on the mechanical stimuli to be examined, that is to what extent static or cyclic forces, sliding forces or strains are exerted (ie ^12^, ^13^, ^31^, ^32^, ^33^). The mechanical bioreactor developed by us is able to exert constantly or intermittently a large number of static and cyclic compressive forces with different load levels and frequencies.^33^ One major finding is that the metabolism of chondrocytes is clearly controlled by the mechanical conditions, wherein the magnitude of the metabolic response is subject to the load frequency, strain and type of loading (eg compression, sliding, continuous, intermittent and static).^7^, ^11^, ^12^, ^13^, ^14^, ^28^, ^29^, ^30^, ^31^, ^32^, ^34^, ^35^, ^36^

In vitro research on OA has been undertaken to screen for drugs, cytokines and growth factors that promote cartilage repair and reduce cartilage destruction. Most in vitro pharmacological models have used IL‐1 to induce cartilage degradation.^37^, ^38^, ^39^, ^40^ However, the similarity of OA‐like changes in these in vitro models to those in humans is limited and most valid with respect to the loss of proteoglycan (PG). Thus, the overall goal of our studies was, using mechanical forces in initially healthy cartilage explants, to cause a disrupted metabolism that strongly resembles the phenotype in OA for pharmacological research. We have developed and described a mechanical loading protocol^29^, ^30^, ^34^, ^35^ that induces OA‐like conditions in initially healthy articular cartilage explants. In the current study, we perform comprehensive biochemical analyses of the time‐dependent alterations and the first pharmacological validation of this new in vitro model using a synthetic inhibitor of matrix metalloproteinases (MMPs).

## 2 MATERIALS AND METHODS

### 2.1 Mechanical loading of cartilage explants

**FIGURE 1:**
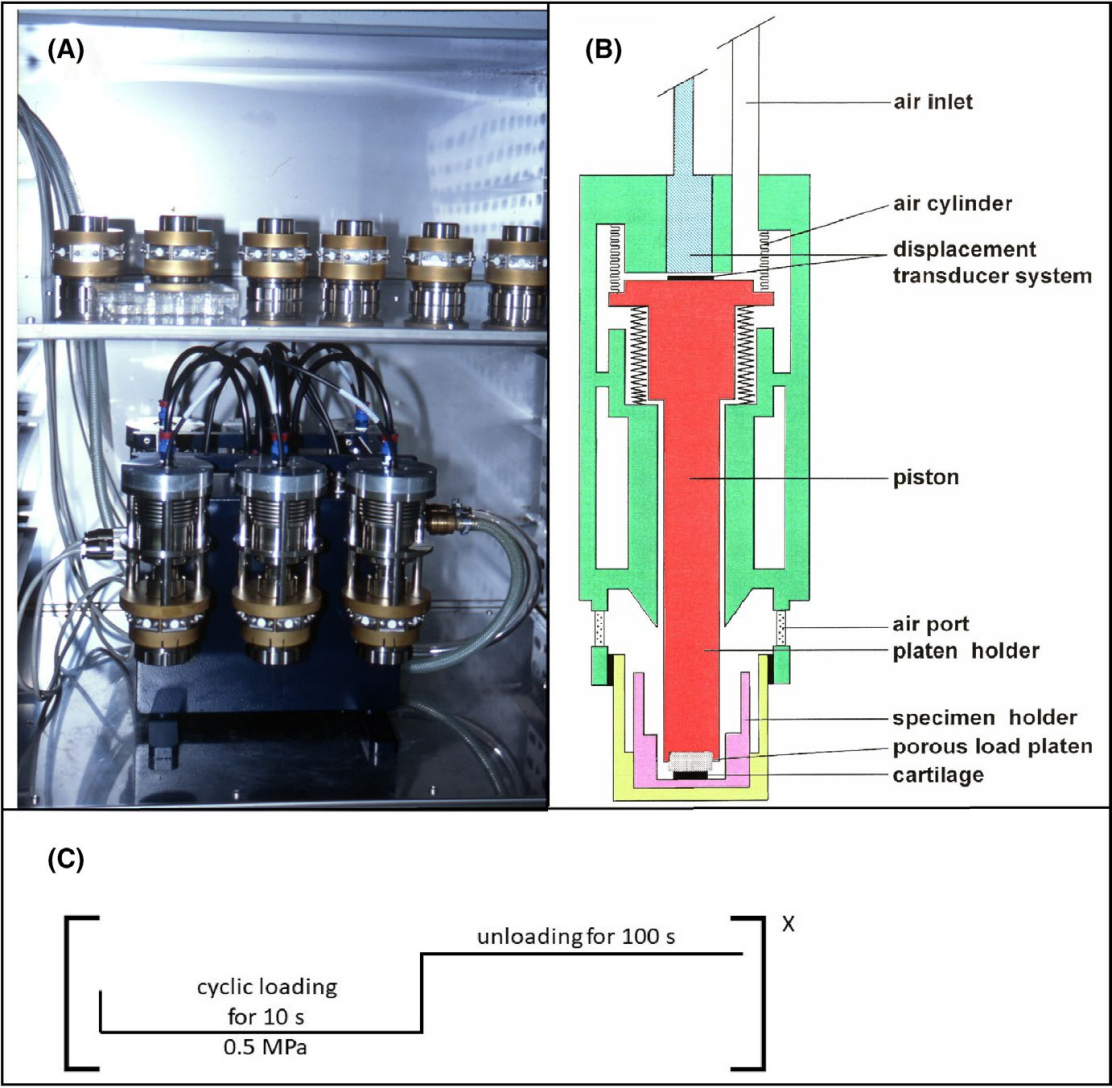
Mechanical loading apparatus. (A) The mechanical device with six load chambers connected to a sealed blue case (bottom) and six control chambers (top) housed within a standard CO_2_ incubator, as described previously.^33^ (B) Schematic of one load chamber, composed primarily of surgical‐grade titanium and stainless steel, with parts thereof being coated with gold‐coloured TiN to make the steel corrosion‐resistant. (C) Schematic representation of the loading protocol. Briefly, explants were intermittent mechanically loaded, in that the loads were cyclically applied in an approximately sinusoidal waveform of 0.1 Hz, followed by a period of unloading lasting 100 s during which the load platen was lifted from the articular surface. This loading sequence was continuously repeated x times until termination of the experiments

To be able to culture and mechanically stress cartilage discs for a longer time period in a sterile surrounding, an apparatus was specially designed and manufactured for this purpose (Figure 1), as described previously.^33^ Briefly, the load amplitude, frequency and load function were controlled by a computerized unit positioned outside of the incubator. Figure 1A shows the six load chambers being mounted in a hermetically sealed case containing a servo‐controlled, electropneumatic valve that regulated the amplitude and frequency of air inlet into the spring‐controlled air bellows. A piston with a porous glass load platen was connected to the dynamic air bellows and mechanically compressed the cartilage explant cultured within a specimen holder (Figure 1B). The specimen holder and piston were made of surgical‐grade titanium. The degree of dynamic cartilage compression was continuously monitored during loading with a load displacement transducer system in the air bellow and the sealed blue case (Figure 1A,B). Eight filter‐controlled air ports per chamber exist to enable under sterile conditions the exchange with the incubator air.

In our study, we used a laminar flow cabinet to obtain two cartilage explants, which were lying side by side and macroscopically healthy, at full‐thickness from the load‐bearing area of a metacarpophalangeal condyle belonging to a slaughtered 18‐ to 24‐months‐old bull. A biopsy punch was used to collect 7 mm diameter cartilage discs. We measured the thickness in the middle of the cartilage explants twice using a digital calliper gauge so that the amount of compression during mechanical loading could be determined.^29^, ^30^, ^34^, ^35^ The discs were washed and centrally located within the specimen holder thereby placing the cutting edge of discs to the bottom of the specimen holders. Articular explants were cultured in 2.5 ml Ham's F‐12 nutrient medium containing a serum substitute and additional supplements as already reported.^29^, ^30^, ^34^, ^35^

The loading device was then equilibrated for 2 h in a normal bench top incubator at 37°C, 5% CO_2_ and 95% humidity (Figure 1A). A peak stress of 0.5 MPa was used to apply uniaxial intermittent cyclic loading for 1, 3, 6 or 9 days. The cyclic loads were applied using a sinusoidal waveform of 0.1 Hz frequency for 10 s, which was followed by a 100‐s period of unloading (Figure 1C). The air inlet was reduced by 10 N during the unloaded period so that the stamp detached from the cartilage surface, enabling the explant to reswell. The cartilage explants serving as controls were cultivated in identically constructed chambers without the air bellows located above. Media were changed on Days 3 and 6 during experiments lasting 6 or 9 days; obtained media received 10% protease inhibitor mixture (vol/vol) and were frozen at −20°C until analysed.

For first pharmacological validation of the in vitro model, the effect of the matrix metalloproteinase inhibitor A976157 [(S)‐2‐(4′‐chloro‐biphenyl‐4‐sulfonyl‐amino)‐3‐(5‐fluoro‐1H‐indol‐3‐yl)‐propionic acid kindly provided by Aventis Pharma Deutschland GmbH, Frankfurt, Germany] on PG and Fn metabolism was examined at a concentration of 10 µM. This MMP inhibitor reduces the activity of MMP‐3 and MMP‐8 with nanomolar IC_50_ values, versus micromolar IC_50_ values against MMP‐1 and MMP‐9. Two cartilage discs lying side by side at the load‐bearing area of one condyle were loaded or unloaded in the presence of the drug. Another set of two explants, which were removed from the same condyle, were loaded or unloaded in the presence of the drug vehicle.

The cultured discs were radioactively labelled with 10 µCi/ml [^35^S]‐SO_4_ or 4 µCi/ml [^14^C]‐proline (both from DuPont de Nemours GmbH, Bad Homburg, Germany) in the presence of 100 µg ml^−1^ ß‐aminopropionitrile or 10 µCi ml^−1^ [^3^H]‐phenylalanine (Amersham Pharmacia Biotech GmbH, Braunschweig, Germany) or 50 µCi ml^−1^ D‐6‐[^3^H]‐glucosamine (Hartmann Analytik GmbH, Braunschweig, Germany) during the last 18 h of the experiments. The experiment was ended by collecting the media and freezing them at − 20°C in the presence of a 10% mixture (vol/vol) of protease inhibitors. GBSS was used to wash three times the cartilage discs before being frozen in GBSS with a 10% mixture (vol/vol) of protease inhibitors at − 20°C until further analysis. The load platen was extracted for 48 h at 4°C using 1 ml of a buffered 4 M guanidinium chloride solution containing 10% (v/v) proteinase inhibitor cocktail and then stored at − 20°C frozen until analysis.

### 2.2 Proteoglycan synthesis

Cartilage discs were thawed, weighed and digested at 65°C for 4 h with 1.0 ml of papain solution (0.5 mg ml^−1^, pH 6.5), which contains 50 mM sodium dihydrogen phosphate, 10 mM EDTA and 2 mM N‐acetylcysteine. [^35^S]‐SO_4_‐radiolabeled PGs in the digested cartilage discs, media and extracts of the load platen were analysed after isolation of macromolecular [^35^S]‐SO_4_‐radiolabeled PGs using PD‐10 columns packed with Sephadex^R^G‐25 resin (Merck KGaA, Darmstadt, Germany), followed by liquid scintillation counting, as described previously.^30^, ^34^

### 2.3 Quantitation of proteoglycans and DNA

The sulphated PGs of the papain‐digested extracts and media were quantified spectrophotometrically at 523 nm after chemical action with 0.25 ml of dimethylmethylene blue using a microplate reader. We used as standard CS type A of bovine trachea (Sigma GmbH, Deisenhofen, Germany).^30^, ^34^ The bisbenzimidazole dye Hoechst 33258 was applied to fluorometrically determine the quantity of DNA in the papain‐digested discs.^34^

### 2.4 Analysis of fine structure of CS

Sulfation of endogenous and [^3^H]‐glucosamine‐labelled CS was monitored in papain‐digested cartilage explants, as described previously.^30^, ^41^ Briefly, papain‐digested tissue samples were centrifuged to remove tissue debris, and GAGs were pelleted, washed, dried, resuspended and digested with chondroitinases ABC and ACII (Seikagaku Corp., Tokyo, Japan). Enzymatically digested CS, containing the unsaturated disaccharides of the internal CS chains, and the nonreducing terminal mono‐ and disaccharides were concentrated using the MicroCon^®^ 3 filters, reduced with NaBH_4_ and injected onto a CarboPac PA1 column that was connected to a guard column (Dionex GmbH, Idstein, Germany). Specimens were eluted by a step gradient and scanned on a HPAEC‐PAD system (model DX 500; Dionex GmbH, Idstein, Germany) to quantify the unlabelled, unsaturated disaccharides ΔDi6S, ΔDi4S and ΔDi0S, obtained from the digested interior part of the CS chains. To quantify [^3^H]‐radiolabelled mono‐ and disaccharides, 1‐ml fractions of the eluant were collected and the radioactivity was quantified by liquid scintillation counting. We calculated the mean length of the newly synthesized CS chains (ie the number of internal Δdisaccharides per chain). For this purpose, the ratio of the radioactively labelled internal unsaturated disaccharides to the terminal saccharides was computed.^41^, ^42^

### 2.5 Fibronectin and protein synthesis

The wet weight of explants was determined after weight equilibrium of thawed discs had been reached. Cartilage and load platen were extracted three times over 2 d for Fn with 4 M urea in 0.05 M phosphate buffer, pH 7.3 and proteinase inhibitors.^35^ [^3^H]‐Phenylalanine‐labelled Fn was isolated from nutrient media and extracts by gelatin Sepharose 4B affinity chromatography.^35^

In addition to Fn, a large number of cartilage proteins were extracted by urea. We determined the total [^3^H]‐phenylalanine incorporated into proteins within the media and urea extracts by precipitation with an identical volume of ice‐cold 1N perchloric acid. After 1 h, the precipitated radioactively labelled proteins were concentrated, washed, and dried and the radioactivity was then determined by liquid scintillation counting.^35^ We determined the Fn content in the extracts and nutrient media of the explants using ELISA.^35^

### 2.6 Collagen synthesis

[^14^C]‐Proline incorporation into collagen was measured using highly specific collagenases as per a modified version of the procedure reported by Peterkofsky and Diegelmann,^43^ as reported previously.^29^ In brief, proteins of lyophilized cartilage explants and media were digested with extremely pure collagenase type VII (Sigma, Deisenhofen, Germany), which was terminated by the addition of 10% (v/v) trichloroacetic acid/0.5% tannin and 20 µl of 2.5% (w/v) bovine serum albumin. After 1 h, the ice‐cold solutions were centrifuged and the radioactivity of digested [^14^C]‐labelled collagen within the supernatant determined by liquid scintillation counting. After the pellet had been washed again, pellets with NCPs were dissolved in 0.2N NaOH and neutralized, after which the radioactivity was determined. We calculated the fraction of collagen among the newly synthesized proteins as per a formula accounting for the enriched presence of collagenous proline in comparison with other proteins.^29^

### 2.7 Water content

We determined the water content of explants gravimetrically, which can provide an indication of possible injury of the extracellular collagenous network. At the completion of the experiments, the cartilage discs were kept in GBSS containing 10% (vol/vol) protease inhibitor mixture for 1.5 h, weighed, lyophilized overnight (Lyovac GT2; Leybold Heraeus GmbH, Cologne, Germany) and weighed again. The difference between wet and dry weight was calculated, and the percentage of water content was then calculated for both unloaded controls and loaded cartilage discs.

### 2.8 Chondrocyte viability

The same culture conditions as described above were applied to cartilage discs that were cultured exclusively to determine the viability of chondrocytes in each cartilage zone separately using the vital stains fluorescein diacetate and propidium iodide.^34^

### 2.9 Statistical analysis

Our experiments were replicated four to five times on different days, each time using joints from different steers (N = 5–6). We analysed the data by one‐ or two‐way analysis of variance (ANOVA), followed by Dunnett's or Šidák‐Holm post hoc test to calculate the multiplicity‐adjusted p‐values for multiple comparisons. In unbalanced experiments with limited missing data, a mixed‐effects model was fitted, followed by Dunnett's post hoc test.

A threshold of *p* ≤ 0.05 was chosen for significance. All data are presented as means and standard deviations. GraphPad Prism^®^ version 8.4.3 for Windows (GraphPad Software, San Diego, CA, USA) was applied for statistical analysis and creation of graphics.

## 3 RESULTS

### 3.1 Biosynthetic activity of load‐free cartilage explants

**FIGURE 2:**
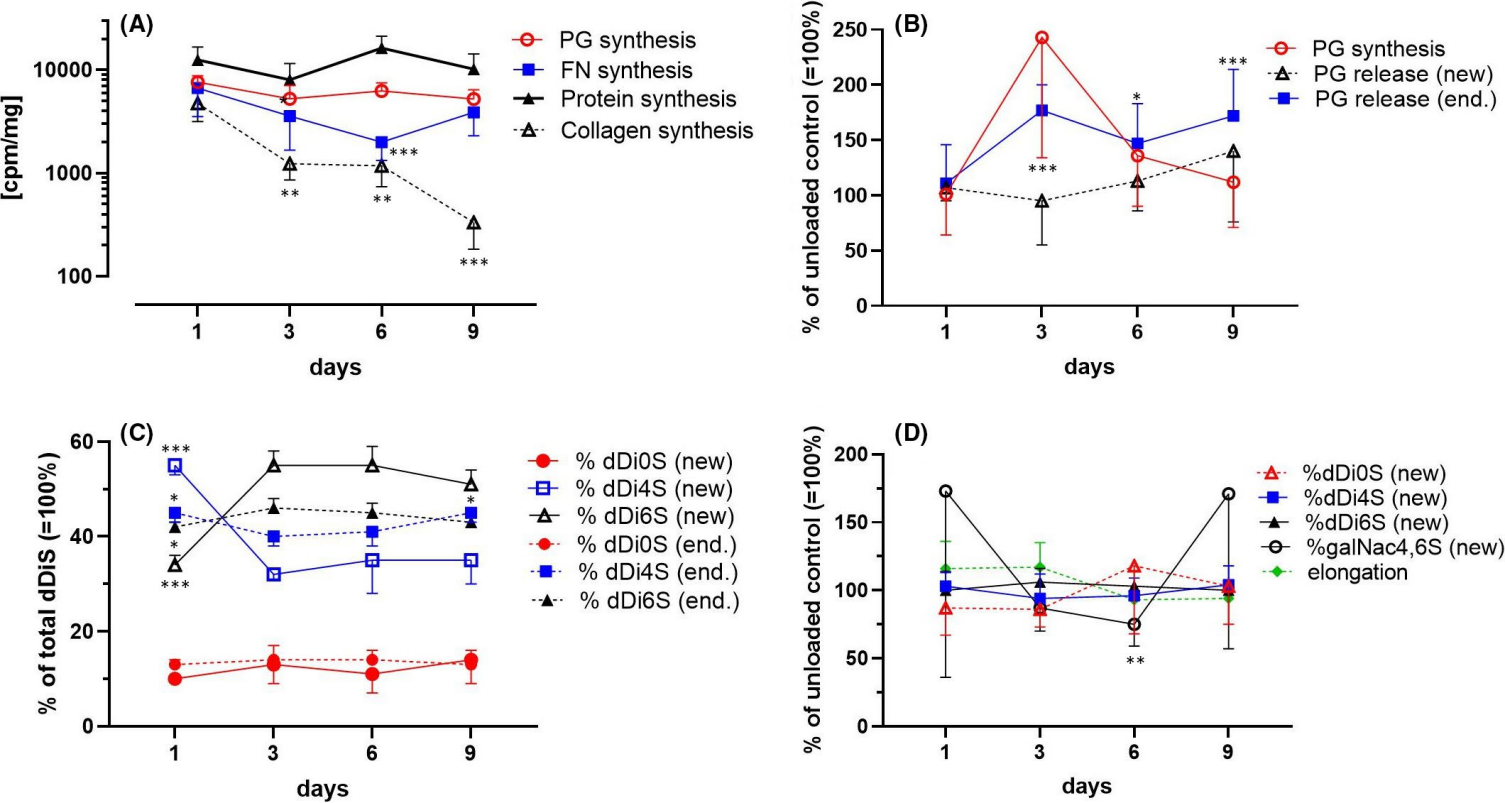
Biosynthetic activity of cartilage explants. (A) Biosynthesis of PG, Fn, protein and collagen by unloaded discs cultured for 1, 3, 6 and 9 days in supplemented Ham's F‐12 media including a serum substitute. (B) Effect of mechanical loading on the synthesis and release of PGs. (C, D) Effect of culture duration (C) and intermittent mechanical loading (D) on the percentage of endogenous and newly synthesized internal disaccharides, elongation and galNAc4,6S termini of CS chains from unloaded (C) or loaded (D) explants. Data were calculated as (A) cpm per mg wet or dry (collagen) cartilage explants, (B, D) percentage of unloaded controls ( = 100%) and (C) percentage of sum of endogenous or newly synthesized disaccharides ( = 100%). Data are presented as means and SD (N = 5–6). The significance threshold was set to *p *≤ 0.05 compared with Day 1 (A), Day 6 (C) or unloaded control values (B, D): ^*^0.01<*p *≤ 0.05, ^**^0.001 < *p* < 0.01, ^***^ *p *≤ 0.001 [Šidák's multiple comparisons following two‐way ANOVA for the effect of loading (B, C); Dunnett's post hoc test following one‐way ANOVA for the effect of culture duration (A, C)]

The synthesis of PGs, fibronectin (Fn) and proteins by unloaded bovine articular discs persisted almost unchanged for up to 9 days of culture (Figure 2A). However, collagen synthesis dropped markedly from Day 1 to Day 9. The chondrocytes in the three histologically discernible zones of explants remained vital over the entire culture period, as determined by fluorescence microscopy in cartilage sections using vital dyes (data not shown).

### 3.2 Proteoglycan metabolism of loaded explants

Intermittent cyclic mechanical loading increased the incorporation of a radioactive precursor into PGs of cartilage explants compared with the case in unloaded controls (Figure 2B). The elevated incorporation appeared after 3 (*p* ≤ 0.001) and 6 days, returning to preload levels by Day 9, indicating a period of maximum mechanical stimulation of PG synthesis. Elevated amounts of endogenous PGs were released into the nutrient media during the 3‐, 6‐ and 9‐day experiments, whereas the total PG content (data not shown) and loss of newly synthesized PGs remained unchanged.

### 3.3 Fine structure of chondroitin sulphate in cartilage explants

The glycosaminoglycan chain chondroitin sulphate (CS) of aggrecan, the main proteoglycan of the cartilage extracellular matrix, is composed out of repeating disaccharides, which themselves consist out of glucuronic acid (glcUA) and ß1,3N‐acetylgalactosamine (galNAc). Analysis of the CS composition being newly synthesized by unloaded explants revealed that culture for up to 9 days did not affect the internal sulfation pattern (Figure 2C). However, during the first 3 days, the 4‐ and 6‐sulfation of newly synthesized and endogenous disaccharides adjusted to the mechanical loading environment and remained constant thereafter. No terminal glcUA, attached to 6‐sulphated galNAc, was identified. In addition, analysis with the high‐performance anion‐exchange chromatography‐pulsed amperometric detection (HPAEC‐PAD) system revealed that the endogenous CS chains had an internal sulfation pattern of disaccharides during the 9 days culture period which was similar to that of newly synthesized CS chains. No alteration of CS chain length, including elongation and shortening, was observed in unloaded explants through the culture (data not shown).

Less galNAc4,6S was synthesized in cartilage discs that were loaded for 6 days, which was the only terminal CS chain that was significantly altered compared with the unloaded controls (Figure 2D). Furthermore, the internal disaccharides of newly produced CS chains and the elongation of these chains remained unaffected by the applied loads.

### 3.4 Fibronectin and protein metabolism of loaded cartilage

**FIGURE 3:**
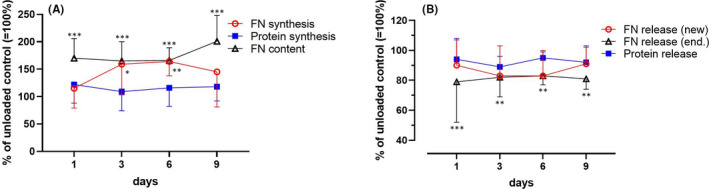
Intermittent mechanical loading of cartilage modulates the synthesis (A) and release (B) of fibronectin and proteins. [^3^H]‐Phenylalanine‐labelled Fn and proteins per mg wet weight of discs and total content of endogenous and radiolabelled Fn (ng/mg wet weight of explant) in extracts of cartilage and load platen as well as in media were determined. Data are percentages of unloaded controls ( = 100%) and are displayed as means and SDs (N = 5–6). The significance threshold was set to *p *≤ 0.05 compared with unloaded controls: ^*^0.01 < *p *≤ 0.05, ^**^0.001<*p* < 0.01, ^***^ *p *≤ 0.001 (Šidák's multiple comparisons following two‐way ANOVA)

The applied loading protocol significantly elevated the incorporation of [^3^H]‐phenylalanine into Fn on Days 3 and 6 compared with that of the unloaded control explants (Figure 3A), again demonstrating a window in which the biosynthetic activity of chondrocytes was enhanced. Protein synthesis, however, remained unchanged; thus, on Days 3 and 6, only the biosynthesis of Fn was specifically stimulated, as the proportion of Fn relative to total newly synthesized protein was increased (data not shown). As shown in Figure 3A, the total endogenous Fn content as found in explants, media and extracts of load platen was also markedly higher, as determined with ELISA. Furthermore, endogenous Fn was less released from loaded explants compared to unloaded controls (Figure 3B).

### 3.5 Collagen synthesis of loaded cartilage

**FIGURE 4:**
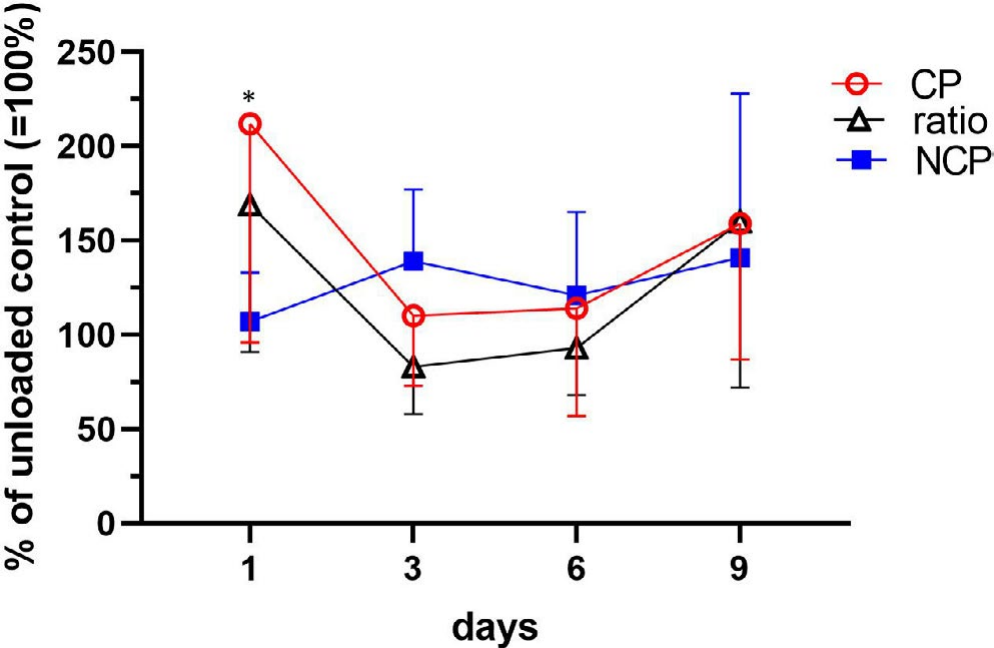
Intermittent mechanical loading modulates the synthesis of collagen. Newly synthesized collagen (CP) and non‐collagenous proteins (NCPs) were determined by the incorporation of [^14^C]‐proline per mg dry weight explant, and the fraction of collagen from NCPs (ratio: relative rate of collagen synthesis) of loaded explants is displayed as a percentage of unloaded control discs ( = 100%). Data are expressed as means and SDs (N = 5–6). The threshold for significance was set to *p *≤ 0.05 versus unloaded control values: *0.01< *p*≤ 0.05 (Šidák's multiple comparisons following two‐way ANOVA)

During the first 3 days of cyclic mechanical stimulation, the incorporation of [^14^C]‐proline, respectively, the specific collagen synthesis rate adapted to the mechanical environment and subsequently remained constant (Figure 4). However, mechanical loading did not modulate the biosynthesis of non‐collagenous proteins (NCP) throughout the 9‐day culture. Furthermore, release of newly synthesized proteins from the loaded cartilage discs into media remained unaffected (data not shown).

### 3.6 DNA and water content of loaded explants

As shown in Table 1, the content of DNA and water from loaded cartilage discs did not differ markedly from values obtained from unloaded explants used as controls.

**TABLE 1:** Effect of loading duration on the DNA and water content and the magnitude of cartilage compression.

Loading duration	No. of specimens	DNA content		Water content		Compression (%)
		Loaded	Unloaded	Loaded	Unloaded	
1 day	6 (38)	0.12 ± 0.02	0.14 ± 0.03	66.5 ± 2.6	67.8 ± 1.8	28.8 ± 7.10
3 days	6 (33)	0.14 ± 0.06	0.12 ± 0.03	69.2 + 4.2	68.1 ± 1.4	30.2 ± 6.89
6 days	6 (35)	0.26 ± 0.04	0.24 ± 0.05	69.5 ± 4.5	67.9 ± 1.7	29.7 ± 7.56
9 days	6 (35)	0.17 ± 0.05	0.15 ± 0.03	70.4 ± 1.7	69.5 ± 2.7	37.9 ± 7.86

Note

Cartilage discs were cyclically mechanically loaded with a maximum pressure of 0.5 MPa, applied in a sinusoidal waveform with a frequency of 0.1 Hz. Discs were subjected to intermittent loading with 10 s of cyclic loading followed by a 100‐s unloading phase repeated x times for 1, 3, 6 or 9 days. Data are shown as mean values ± S.D. The numbers in parentheses indicate the numbers of specimens used to quantify the degree of compression.

### 3.7 Load induced compression

The extent of the maximum compression of the mechanically loaded cartilage discs remained constant throughout the experiments (Table 1). As shown in Table 1, the range of standard deviations was ± 7.3%. This implies that the on average 31.6% load‐induced compression of cartilage discs, which were originally 0.54 ± 0.05 mm thick (N = 141), was reproducible under the respective conditions.

### 3.8 Viability of chondrocytes

Staining of the sections of loaded explants with vital dyes revealed that the applied mechanical pressure was harmful to the superficial zone of cartilage discs, resulting in significantly fewer living chondrocytes [percentage of living cells on Days 1, 3, 6 and 9, normalized to control ( = 100%): 37.4 ± 29.4%, 25.3 ± 11.8%, 4.2 ± 2.3% and 5.1 ± 7.1%, respectively]. This deleterious impact was most pronounced in cartilage discs that were mechanically stressed for 9 days. Cells of the intermediate and deep zones remained vital and were not affected (data not shown).

### 3.9 First pharmacological validation of the in vitro model

The suitability of the in vitro model for pharmacological research was tested in that the effect of the MMP‐inhibitor A976157 on PG and Fn metabolism of cartilage explants was examined at 10^−5^ M in a 6‐day experiment.

**FIGURE 5:**
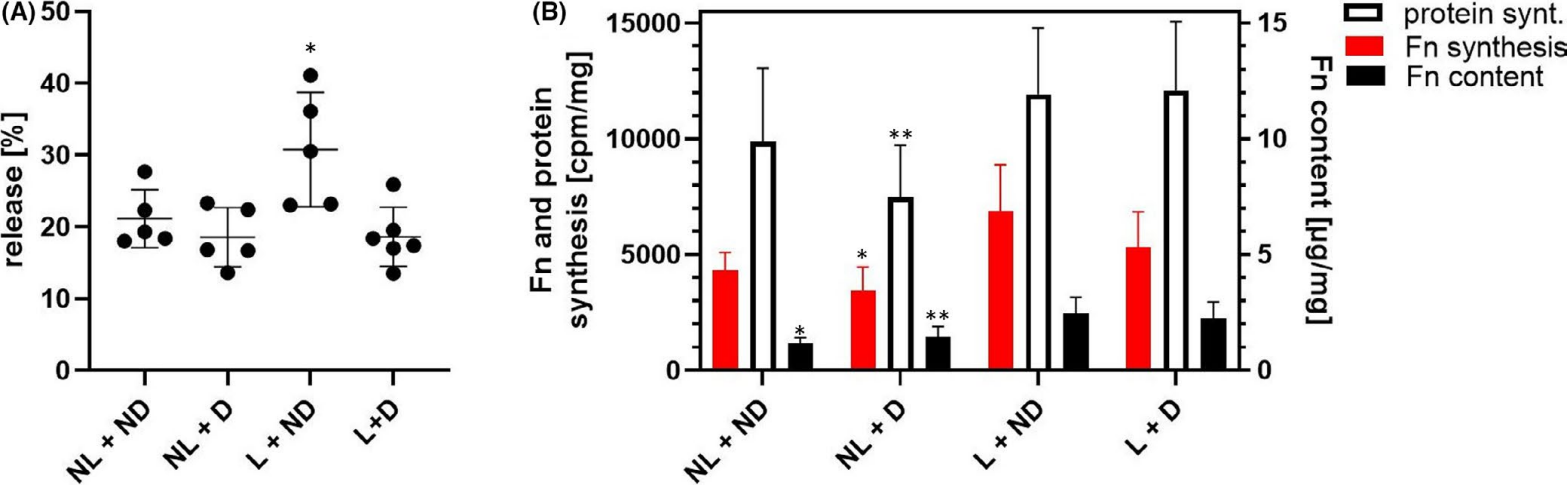
Effect of the MMP inhibitor A976157 on mechanically induced release of PGs (A) and synthesis of fibronectin (B) and proteins by bovine cartilage discs. The release of endogenous and radiolabelled PGs into media per µg DNA is expressed as percentage of total PGs in cartilage, nutrient medium and load platen extract. The incorporation of [^3^H]‐phenylalanine into Fn and proteins per mg wet weight of discs and the total content of Fn (µg/mg cartilage) in extracts of cartilage and load platen as well as media are presented. Intermittent cyclic loading was applied to explants for 6 days. Data are expressed as means and SDs (N = 5–6). The significance threshold was set to *p *≤ 0.05 versus loaded explants treated with A976157: ^*^0.01<*p *≤ 0.05 (mixed‐effects analysis followed by Dunnett's post hoc test versus L + D). NL: No Load; ND: No Drug; L: Load; D: Drug (MMP inhibitor)

The MMP inhibitor led to significant reversal of the mechanically induced release of endogenous PGs to control levels (Figure 5A). However, the agent did not modify the biosynthesis or total content of PGs, DNA content, the maximum amplitude of compression of explants or the release of newly synthesized PGs (data not shown).

Figure 5B shows that the load‐induced increases in the biosynthesis and content of Fn and protein synthesis were not significantly affected by A976157. However, as expected, mechanical loading of drug‐treated explants increased the synthesis of Fn and protein as well as Fn content. In addition, the release of Fn and proteins remained unaffected (data not shown).

Cartilage sections were dyed with the vital stains propidium iodide and fluorescein diacetate, revealing that A976157 did not impair chondrocyte viability (data not shown).

## 4 DISCUSSION

In humans, secondary OA has a largely mechanical aetiology, and in many animal models, OA can be artificially induced by mechanical instability.^15^, ^16^, ^17^, ^19^ This in vitro study revealed the time dependence of the mechanically induced changes of our new in vitro model, developed for the preclinical testing of drugs against OA, as well as the first pharmacological validation of it.

The loading device that we used^33^ was constructed to load cartilage explants perpendicularly to the cartilage surface by radially unconfined compression, instead of controlling the hydrostatic pressure or the displacement of explants. Cartilage discs were loaded on the day of slaughter because our experiments with unloaded explants showed that the biosynthetic activity with respect to PG, protein and Fn remains constant in vitro for at least 9 days. The bovine knee joints were found to have static compressive stress of around 0.8 MPa, and constant stresses on cartilage surfaces of differently sized species were reported.^44^ The pressure in our study was set to 0.5 MPa, which is below the stresses experienced by the human knee joints while walking (0.8–6.3 MPa).^45^ Although this pressure is low, the strains in our experiments were above those that are expected to occur in vivo because the cartilage discs were removed from the subchondral bone and its enclosing cartilage. Moreover, cartilage discs were obtained from bovine metacarpophalangeal joints and not from human knee or hip joints. However, our previous studies identified the frequency of cyclic loading as the chief mechanical factor that influences cartilage metabolism.^29^, ^30^, ^34^, ^35^

In this study, we determined a significantly enhanced loss of endogenous PGs from cartilage discs after 3 days of loading. A distinctive feature of OA is the depletion of PGs from damaged joint cartilage.^46^, ^47^ Our findings are corroborated by earlier in vitro studies also reporting mechanical load‐induced damage of the collagenous network, leading to an enhanced loss of PGs.^48^

Consistent with earlier results,^49^, ^50^, ^51^ we observed greater release of PGs only after a latent period, perhaps due to the production and activation of proteoglycanolytic enzymes, which could have disrupted the balance between MMPs and TIMPs, their physiological inhibitors. The synthetic, non‐peptide MMP inhibitor A976157 suppresses the activity of MMP‐3 and MMP‐8 with IC_50_ values in the nanomolar range. Both MMPs are synthesized by chondrocytes and cleave PGs in the interglobular domain between Asn^341^ and Phe^342^.^52^, ^53^, ^54^ In our experiments, we observed nearly complete inhibition of the release of PGs by the MMP inhibitor, implicating a key role of MMPs in the degradation of PGs.

Correlating with the greater load‐induced loss in PG, chondrocytes synthesized more PGs, which reached significance after 3 and 6 days. This increased biosynthesis of PGs might constitute a chondrocytic response to the mechanical damage of cartilage tissue with the aim of stabilizing the weakened extracellular matrix. Similarly, higher PG biosynthesis was reported for a cruciate ligament model of early OA, which was interpreted as an attempt to repair damaged tissue.^55^

Our loading protocol significantly decreased the nascent 4,6‐disulfation of the nonreducing terminal galNAc residue, whereas the ratio of ΔDi6S to ΔDi4S of the endogenous CS chains remained constant. In human OA cartilage, less than 40%–50% of the CS chain termini are galNAc4,6S, whereas the ratio of ΔDi6S to ΔDi4S in CS chains is unaltered or lower in human articular cartilage in OA.^42^, ^56^, ^57^ The above‐mentioned terminal galNac4,6S was newly synthesized apart from the 6‐sulfation of the internal disaccharides and confirmed our earlier reports.^30^, ^41^ These divergent effects of mechanical impact on the molecular composition of CS chains with respect to the OA‐like appearance require further examination to determine the biosynthetic response mechanisms of chondrocytes.

Our loading protocol also significantly increased Fn synthesis after a latent period of 3 and 6 days, whereas protein synthesis remained unchanged. Several studies have confirmed that OA cartilage synthesizes up to five times more Fn than healthy cartilage, that Fn levels are 10‐fold to20‐fold higher in early OA cartilage lesions and that OA cartilage explants release less newly synthesized Fn into media.^58^, ^59^, ^60^ We also observed significantly greater retention of endogenous and newly synthesized Fn in loaded explants. Our results are supported by previous studies that demonstrated higher synthesis and concentration of Fn in canine cartilage discs ten days post intermittent impact pressure.^61^, ^62^

Any load‐induced increases in Fn synthesis and content remained unaffected by the addition of the MMP inhibitor A976157. MMP‐3 and MMP‐8 were reported to cleave Fn,^63^ and a single blunt impact on bovine osteochondral explants was found to promote Fn fragmentation.^64^ Thus, the MMP inhibitor might have reduced the MMP‐3‐ and MMP‐8‐generated Fn fragments in cartilage explants, perhaps antagonizing articular cartilage degradation, because these fragments induced the expression of destructive MMPs in vivo by chondrocytes and synoviocytes, including MMP‐1, MMP‐3, MMP‐8 and MMP‐13.^64^, ^65^ However, additional experiments are needed to confirm the inhibition of load‐induced Fn fragmentation by an MMP inhibitor.

Mechanical loading of explants did not alter collagen synthesis for up to 9 days. After skeletal growth, the biosynthesis of collagen type II decreased significantly, and by in situ hybridization and immunohistochemistry, collagen type II was reported to be not expressed in normal human knee cartilage.^66^, ^67^ However, in early OA cartilage, the expression of mRNA collagen type II is robust, especially in chondrocytes from the middle zones, which was supported by the higher level of C‐propeptide of type II procollagen found in human OA cartilage.^66^, ^67^ These findings are consistent with those using adult canine cruciate ligament model of OA, in which collagen synthesis increased up to 10‐fold within 2 weeks after surgical joint injury.^68^ Although the rise in collagen synthesis can be interpreted as an attempt of OA chondrocytes to replace impaired extracellular matrix, Garnero et al. suggested that collagen type II synthesis is uncoupled from degradation.^69^ Collectively, our findings indicate that the unaltered collagen synthesis by bovine explants is related to this uncoupling effect and to the minor damage of the collagen network, such as cracks in the cartilage surface.

Thus, it is unexpected that the water content between loaded and unloaded control discs was similar. Elevated water content of up to 9% in human femoral head cartilage belong to the early alterations seen in human and experimentally induced animal OA.^70^ Furthermore, in vitro studies revealed that the water content of cartilage explants increases after impact loading.^61^, ^62^, ^71^ These findings suggest irreversible weakening of the retaining collagen network, wherein hydrophilic PGs swell by hydration to a greater degree than normal. Again, our data indicate that the collagen network of explants is not damaged severely enough for an increase in water content to be quantifiable.

During OA, the articular cartilage surface loses its homogeneous nature and is disrupted and fragmented by pitting, clefts and ulcerations.^72^, ^73^ In our experiments, the porous load platen slightly damaged the cartilage surface and thus contributed to the induction of OA‐like changes. A porous load platen was chosen to ensure that the explants were adequately supplied with nutrients.

In conclusion, a notable feature of our new in vitro model is the induction of an early‐stage OA‐like appearance in response to purely mechanical stimuli, which, when in excess, is a major pathogenic factor of human OA. Our first pharmacological prevalidation already showed that this new biomechanical in vitro model is suitable to screen potentially chondroprotective agents. Compared with animal models of OA, nearly every biochemical, histological or biomechanical method can be applied in our novel in vitro model within a reasonable period. Thus, our novel biomechanical in vitro model should deepen our understanding of the early stages of degenerating cartilage and its specific treatment.

## CONFLICT OF INTEREST

The authors declare no competing or financial interests. Manfred Schudok is a Sanofi employee and may hold shares and/or stock options in the company.

## AUTHOR CONTRIBUTION

**Katrin Sauerland:** Data curation (equal); Formal analysis (equal); Investigation (equal); Methodology (equal); Writing‐review & editing (equal). **Amela Wolf:** Data curation (equal); Formal analysis (equal); Investigation (equal); Methodology (equal); Writing‐review & editing (equal). **Manfred Schudok:** Formal analysis (equal); Investigation (equal); Methodology (equal); Project administration (equal); Resources (equal); Writing‐review & editing (equal). **Juergen Steinmeyer:** Conceptualization (lead); Data curation (equal); Formal analysis (equal); Funding acquisition (lead); Investigation (equal); Methodology (equal); Project administration (lead); Supervision (lead); Writing‐original draft (lead); Writing‐review & editing (equal).

## Data Availability

The data supporting the findings of this study are available from the corresponding author upon reasonable request.
